# Factors impacting survival in individuals with Down syndrome‐associated Alzheimer's disease

**DOI:** 10.1002/alz.71156

**Published:** 2026-02-17

**Authors:** Bessy Benejam, Mary McCarron, María Carmona‐Iragui, Pamela Dunne, Lucia Maure‐Blesa, Miren Altuna, Javier Arranz, Isabel Barroeta, Alexandre Bejanin, Laura Del Hoyo Soriano, Susana Fernández, Sandra Giménez, Alberto Lleó, Louise Lynch, Philip McCallion, Niamh Mulryan, Lucia Pertierra, Anne‐Sophie Rebillat, Íñigo Rodríguez‐Baz, Aida Sanjuan Hernández, Lídia Vaqué‐Alcázar, Laura Videla, Andrew Wormald, Juan Fortea, Eimear McGlinchey

**Affiliations:** ^1^ Sant Pau Memory Unit, IR SANT PAU Hospital de la Santa Creu i Sant Pau Barcelona Spain; ^2^ Barcelona Down Medical Center Fundació Catalana Síndrome de Down Barcelona Spain; ^3^ Trinity Centre for Ageing and Intellectual Disability School of Nursing and Midwifery Trinity College Dublin Dublin Ireland; ^4^ Avista CLG Clonsilla Dublin Ireland; ^5^ Center of Biomedical Investigation Network for Neurodegenerative Diseases (CIBERNED) Madrid Spain; ^6^ Department of Medicine Universitat Autònoma de Barcelona (UAB) Bellaterra Spain; ^7^ Center for Research and Memory Clinic CITA‐Alzheimer Foundation Donostia‐San Sebastián Spain; ^8^ Department of Neurology Bioaraba Health Research Institute Araba University Hospital‐Txagorritxu Vitoria‐Gasteiz Spain; ^9^ Multidisciplinary Sleep Unit Respiratory department Hospital de la Santa Creu i Sant Pau Institut d'Investigació Biomèdica Sant Pau (IIB SANT PAU) Barcelona Spain; ^10^ School of Social Work Temple University Philadelphia Pennsylvania USA; ^11^ Institute Jerome Lejeune Paris France; ^12^ Department of Medicine Faculty of Medicine and Health Sciences Institute of Neurosciences University of Barcelona Barcelona Spain; ^13^ Department of Psychobiology Institut d'Investigacions Biomèdiques August Pi i Sunyer (IDIBAPS) Barcelona Spain; ^14^ Global Brain Health Institute Trinity College Dublin Dublin Ireland

**Keywords:** Alzheimer's disease, Down syndrome, Down syndrome‐associated Alzheimer's disease, LOMEDS, mortality, survival

## Abstract

**INTRODUCTION:**

Adults with Down syndrome (DS) are at high risk for Alzheimer's disease (AD), the leading cause of death in this population. Survival in DS after AD diagnosis appears shorter than in sporadic AD; however, the factors influencing survival remain poorly understood.

**METHODS:**

We analyzed 157 adults with DS from Spain and Ireland who died of AD between 2012 and 2024. Clinical, genetic, and care predictors were examined using Kaplan–Meier curves and Cox regression.

**RESULTS:**

Mean survival after AD diagnosis was 4.8 years (SD 3.5). Those in specialist intellectual disability dementia care had a longer survival time (mean 9.5 years) than other settings (mean 3.4 to 4.1 years; *p* < 0.001). Late‐onset myoclonic epilepsy in DS (LOMEDS) was linked to a threefold higher risk of death after onset (*p* < 0.001).

**DISCUSSION:**

Specialist care settings and LOMEDS timing significantly shape survival in DS‐associated AD, highlighting the importance of tailored services and proactive epilepsy treatment.

## Highlights

- Mean survival after DSAD dementia diagnosis in a multicenter cohort was 4.8 years.
- People with DS in specialist ID dementia care lived the longest, 9.5 years.
- People with DS in community, residential, or nursing care facilities lived 3.4 to 4.1 years.
- LOMEDS was associated with a threefold higher risk of death after onset (HR 3.37).
- Findings support specialist ID and dementia care and support.

## 1 INTRODUCTION

Down syndrome (DS), caused by the trisomy of chromosome 21, represents the most prevalent genetic cause of intellectual disability (ID). Significant advancements in medical care have led to a remarkable increase in the life expectancy of individuals with DS, from approximately 5 to 12 years in the mid‐20th century to around 60 years in recent decades.^1^, ^2^ This increased longevity has, however, brought to the forefront Alzheimer's disease (AD) as the major health concern and the primary cause of morbidity and mortality among older adults with DS.^3^, ^4^

The strong genetic link between DS and AD is well established: The triplication and overexpression of the amyloid precursor protein (APP) gene, located on chromosome 21, results in the overproduction of amyloid beta (Aβ), a critical component in AD neuropathology. Consequently, neuropathological hallmarks of AD, such as Aβ plaques and neurofibrillary tangles, are almost universally observed in the brains of individuals with DS by age 35 to 40, classifying DS as a genetically determined form of AD.^5^

Previous studies have found that survival following an AD diagnosis is notably shorter in DS‐associated AD (DSAD) than in sporadic AD, with median survival times ranging from 3.8 to 4.81 years,^6^ and a mean age at death around 58‐60 years.^7^ This significantly shorter survival highlights AD's limiting effect on longevity in DS^8^.

AD in DS is nearly fully penetrant and has a predictable age of onset, with a mean age of AD dementia diagnosis between 53 and 55 years.^3^, ^8^ However, as in autosomal dominant AD (ADAD), there remains considerable variability,^8^, ^9^, ^10^, ^11^ and the age of dementia diagnosis can extend from individuals under 40 to over 70 years of age.^8^ The cumulative incidence of AD dementia in DS increases exponentially with age, reaching over 95% in the seventh decade of life.^12^ A range of factors have been associated with either an earlier dementia diagnosis or increased mortality risk in people with DS. Individuals living with family are often diagnosed earlier, likely due to greater continuity of care and caregiver familiarity with baseline functioning, factors associated with longer post‐diagnosis survival.^6^, ^7^ Some studies suggest potential benefits of anti‐dementia medication, such as cholinesterase inhibitors, in mortality risk reduction.^13^ The apolipoprotein E (APOE) ε4 allele has been linked to an earlier onset of symptomatic AD in DS and a more rapid progression to death following dementia diagnosis compared to non‐carriers.^14^, ^15^ Late‐onset myoclonic epilepsy in Down syndrome (LOMEDS) commonly co‐occurs with dementia in DS and has been associated with worse cognitive and functional outcomes. Its role in survival and mortality is less clear.^16^, ^17^, ^18^

**RESEARCH IN CONTEXT**

1. **Systematic review**: Existing literature has shown that survival following Alzheimer's disease (AD) diagnosis in individuals with Down syndrome (DS) is shorter than in sporadic AD, but evidence on predictors of survival remains scarce. Prior reports highlighted age at AD diagnosis, severity of intellectual disability, APOE ε4 status, and comorbidities as possible contributors, though results have been inconsistent.
2. **Interpretation**: In this multicenter study of 157 adults with DS who died from AD, survival averaged 4.8 years. Residence in a specialist intellectual disability and dementia care setting was associated with nearly doubled survival, whereas early onset of LOMEDS conferred a threefold increased mortality risk. Neither multimorbidity nor APOE status predicted survival.
3. **Future directions**: Larger prospective studies are needed to confirm the impact of specialized care and to clarify the biological mechanisms linking LOMEDS timing to mortality. Clinical trials exploring targeted epilepsy management may inform future strategies to extend survival in DS‐associated AD.

While many studies have explored risk factors for the onset of DSAD, less is known about the factors that influence survival after diagnosis. Understanding clinical, genetic, and care‐related contributors to survival is essential for improving post‐diagnostic support, informing care strategies and medical guidelines, and guiding prognosis. Care for adults with DSAD can occur across diverse settings, including family homes, small community‐based residences, and larger congregate or specialist facilities. These settings differ in medical oversight, dementia‐specific expertise, and access to end‐of‐life care, all of which may influence survival outcomes. Within this study, we included three cohorts that together capture this diversity: two cohorts (one in Spain, one in Ireland) that included residential, community and family care settings, and one cohort representing a unique specialist residential service that provides dedicated dementia care for adults with intellectual disabilities (in Ireland). This design allowed comparison of survival patterns between heterogeneous cohorts with a mix of typical care settings to a single cohort with a specific model of care.

The aims of this study were twofold: (1) to examine clinical and medical comorbidities, including LOMEDS, APOE genotype, and common health conditions, associated with survival from AD diagnosis to death, and (2) to investigate whether the type of care setting was associated with survival outcomes. We hypothesized that individuals residing in a specialist ID and dementia care setting would demonstrate longer survival compared to those in general or mixed care settings.

## 2 METHODS

### 2.1 Study design and sample

This study included data from individuals with DS who died due to AD between 2012 and 2024 from three distinct cohorts of adults with DS from Spain and Ireland:

**Cohort 1: DABNI –** Individuals from the Alzheimer‐Down Unit of the Catalan Down Syndrome Foundation and the Hospital of Sant Pau, Barcelona, Spain, which integrates a population‐based health plan designed for screening for medical comorbidities with a particular focus on AD. In this cohort, participants are referred to semi‐annual or annual structured neurological and neuropsychological assessments performed by clinicians specializing in DSAD. The Barcelona cohort is also the clinical pillar of the Down Alzheimer Barcelona Neuroimaging Initiative (DABNI), a longitudinal multimodal biomarker project to advance clinical research in DSAD.^19^ Participants from DABNI resided across family, community, and residential care settings.

**Cohort 2: Avista –** Individuals with DS from Avista, residing in a residential care setting designed specifically to support people with ID and dementia, in Dublin, Ireland. The service is supported by a multidisciplinary team and provides person‐centered, long‐term residential care in two specially built homes (an eight‐bed and a six‐bed home) for individuals with mid‐ to late‐stage dementia. Structured health and neuropsychological assessments are conducted annually led by an Advanced Nurse Practitioner in dementia as part of routine care. Further details can be found elsewhere.^12^

**Cohort 3: IDS‐TILDA –** Individuals with DS from the Intellectual Disability Supplement to the Irish Longitudinal Study on Ageing (IDS‐TILDA). IDS‐TILDA is a nationally representative longitudinal observational study on aging in people with ID in Ireland that collects data across a broad range of health and social aspects. The data included were from the subsample of participants with DS, residing across family community and residential care settings. They were collected prospectively via structured interviews and questionnaires completed by participants with ID and/or their caregivers.^20^, ^21^

All cohorts included individuals across all levels of ID and contributed a consistent set of variables, including level of ID, age at AD dementia diagnosis, age at first AD symptom, age at death, comorbidities (including LOMEDS, hypothyroidism, sensory conditions, psychiatric conditions, and hypertension), care setting at diagnosis and death, and AD medication use. Across cohorts, variables were harmonized to indicate the presence of a clinician‐diagnosed condition (yes/no), with dates of diagnosis extracted from the clinical or medical record when available.

In DABNI and Avista, dementia was diagnosed clinically based on the presence of cognitive decline that interfered with daily functioning, as assessed by experienced clinicians specializing in DSAD. In both cohorts, diagnosis was made following clinical and neuropsychological evaluation and following a consensus diagnosis. In IDS‐TILDA, an AD dementia diagnosis was made by a physician, documented in the participant's medical record, and extracted from information provided during the caregiver or family interview. In Avista and DABNI, age at first AD symptom is based on first AD symptom as reported by caregiver. This is recorded by a clinician during interview with the primary caregiver, following DSAD best practice. First symptom was not available for IDS‐TILDA.

Comorbidities were diagnosed in DABNI and Avista in a clinical setting. In IDS‐TILDA, comorbidities were taken from participants’ medical records obtained during an interview, with dates recorded where available. In relation to LOMEDS, diagnoses were made by site clinicians in DABNI and Avista and were defined as an adult‐onset epilepsy syndrome unique to people with DS characterized by corticogenic myoclonic jerks, often upon waking, plus generalized tonic‐clonic seizures associated with the development of symptomatic AD. In IDS‐TILDA, LOMEDS was recorded when new‐onset myoclonic epilepsy, occurring after dementia diagnosis in individuals without prior epilepsy, was documented in the medical record and reported at follow‐up. Data on the APOE ε4 allele were included only from the DABNI cohort.

**FIGURE 1:**
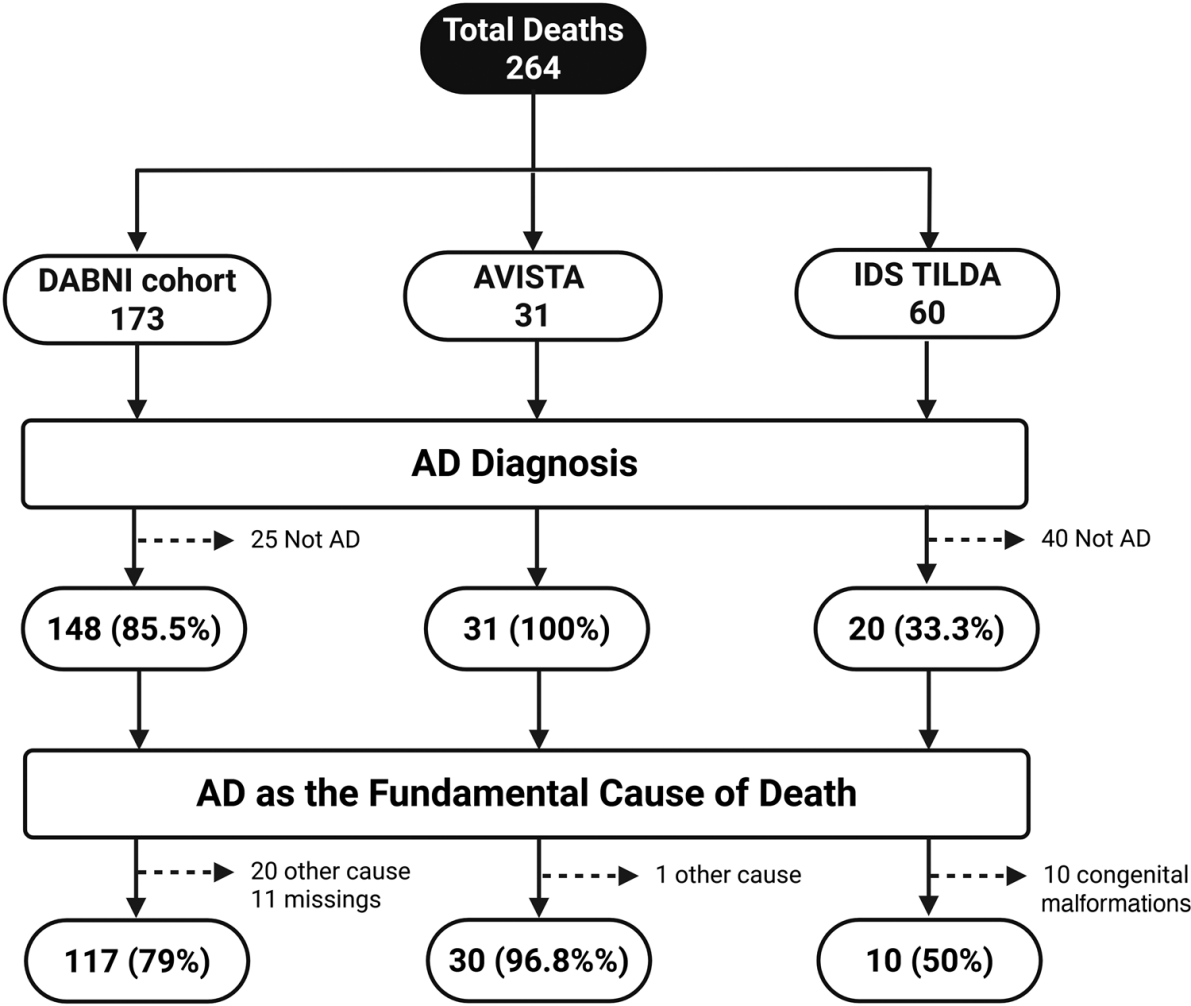
Flow chart for final study analytic sample. Flow chart showing how decedents from each cohort enter the analytic sample. Step 1 screens for an AD dementia diagnosis. Step 2 verifies AD as the fundamental cause of death. Solid arrows indicate inclusion; dotted arrows indicate exclusions such as non‐AD diagnoses, alternative causes of death, or missing cause‐of‐death data. Rounded boxes represent cohorts and the final analytic sample; rectangular boxes are decision nodes. Abbreviations: AD, Alzheimer's disease; ID, intellectual disability.

To define the analytic sample for this study, we identified individuals who had a confirmed clinical diagnosis of AD and for whom AD was recorded as the underlying cause of death. The underlying cause of death was defined using the International Statistical Classification of Diseases and Related Health Problems, 10th Revision (ICD‐10), as the disease or injury that initiated the train of morbid events leading directly to death.^22^ Individuals who died from causes unrelated to AD were excluded. All subsequent analyses to explore the factors influencing survival in DS were performed in the 157 individuals with DS in whom the underlying cause of death was attributed to AD. A flow diagram illustrating the sample derivation is presented in Figure 1.

### 2.2 Statistical analysis

Statistical analyses were performed using IBM SPSS Statistics version 29.0.2.0 and R version 4.5.0. Descriptive statistics were used to summarize the sample, with means and standard deviations reported for continuous variables and frequencies and percentages for categorical variables. The primary outcome was survival time, defined as the number of years between diagnosis of AD dementia and recorded death.

Independent‐samples *t*‐tests and one‐way analysis of variance (ANOVA) were used to compare continuous variables across groups where appropriate. Post hoc comparisons were conducted using Tukey's HSD where applicable. Univariate Cox proportional hazards regression analyses were then conducted for all predictors to estimate unadjusted hazard ratios (HRs) and 95% confidence intervals (CIs). These models were run in the combined sample and separately within each cohort. Multivariable Cox proportional hazards regression was used to estimate adjusted HRs for prespecified predictors, including care setting and selected clinical covariates. For care setting, pairwise differences between all categories were examined. Kaplan–Meier survival curves are presented to illustrate survival patterns. Median and mean survival times with 95% CIs were reported.

### 2.3 LOMEDS analysis

To examine the impact of LOMEDS, we computed the variable “LOMEDS latency” as years from age at AD symptom onset (available only for DABNI and Avista and defined as the age at which the first cognitive or behavioral symptoms attributed to AD emerged) to LOMEDS onset, and for the LOMEDS subanalysis survival time was similarly taken as years from age at AD symptom onset (time zero) to death. Because LOMEDS could occur at different follow‐up times, and to avoid immortal time bias, we fit a time‐dependent Cox proportional hazards model. A counting‐process dataset was created in R 4.5.0 (survival package version 4.4‐0). For participants who developed LOMEDS, two risk intervals were generated: pre‐onset and post‐onset. Those who never developed LOMEDS contributed a single interval. Person‐time was assigned to the pre‐LOMEDS state until LOMEDS onset, and, if applicable, to the post‐LOMEDS state thereafter. The exposure term was a binary indicator switching from 0 to 1 at LOMEDS diagnosis. Latency (years from age at AD symptom onset to LOMEDS) was modeled as a continuous time‐dependent covariate, set to 0 before onset and the observed latency value after onset. Model adequacy was assessed with Schoenfeld residuals; there was no evidence of proportional hazards violation (global test *p* = 0.52). Concordance was 0.65, indicating moderate discrimination. To display model‐based survival, we plotted predicted survival curves from the fitted time‐dependent Cox model for four hypothetical latency scenarios: no LOMEDS, onset at 1 year, onset at the cohort median latency (2.9 years), and onset at 5 years. These scenario times (0, 1, 2.9, 5 years) were prespecified to bracket the empirical latency distribution. For each time, we evaluated the fitted time‐dependent Cox model at that fixed onset and computed the corresponding survival function. The resulting curves are counterfactual, model‐based predictions and do not imply that any participant's latency equaled those exact values.

### 2.4 Predictors of survival from AD dementia diagnosis to death

To assess independent predictors of survival from AD dementia diagnosis to death, multivariate Cox proportional hazards regression analyses were conducted. Avista was excluded from this model due to its unique profile as a specialist ID and dementia care setting with almost all female participants and substantially longer survival times, to ensure estimates reflected typical care environments. Variables included in the model were age at AD dementia diagnosis, sex, level of ID, care setting at the time of death, multimorbidity, timing of LOMEDS, and use of AD medication. All variables were entered simultaneously. Proportional hazards assumptions were assessed and met. HRs and 95% CIs are reported. All statistical tests were two‐tailed, and significance was set at *p *< 0.05.

## 3 RESULTS

### 3.1 Cause of death

The combined dataset across the three cohorts included 264 individuals with DS who died between 2012 and 2024. There were important differences in the percentage of deaths attributed to AD across the three cohorts: 79% in DABNI, 96.8% in Avista, and 50% in IDS‐TILDA. These variations likely reflected both differences in how the cause of death was recorded and differences in the underlying populations. In Avista, individuals died within a specialist ID and dementia care setting, and the cause of death was recorded directly at the time of death, providing accurate and immediate documentation. However, as Avista is a residential setting specifically for those with dementia, the high rate of AD as a cause of death reflects the nature of the setting and does not provide a generalizable estimate of AD‐related mortality. In contrast, DABNI relied on medical records, and IDS‐TILDA drew on public death certificates, which often lack clinical detail and thus likely underestimate AD‐related deaths.

### 3.2 Demographic and clinical profile of sample

Table 1 presents the demographic, clinical, and care‐related characteristics of the overall sample (*n* = 157). The mean age at AD dementia diagnosis was 53.9 years (SD = 3.5, range = 40.9 to 69.2), with a mean age at death of 58.8 years (SD = 6.6) and a mean survival time from diagnosis to death of 4.8 years (SD = 3.5). The sample included slightly more females (58%) than males (42%). Most participants were classified as having moderate ID (56.6%), and comorbid health conditions were common, particularly LOMEDS (73.2%) and hypothyroidism (49%). At the time of AD dementia diagnosis, just over half (51.9%) were living at home with relatives. At the time of death, care settings were more diverse, with 37.3% still at home, 23.9% in ID residential services, and 21.1% in a specialist ID and dementia home. Overall, 57.1% of the sample had taken AD medication since diagnosis.

**TABLE 1:** Sample characteristics of total sample and by cohort: descriptive statistics for demographic, clinical, care setting, and comorbidity variables shown for the total sample and for DABNI, Avista, and IDS‐TILDA.

Variable	Total sample (*n* = 157)	DABNI (*n* = 117)	AVISTA (*n* = 30)	IDS‐TILDA (*n* = 10)
**Age of AD dementia diagnosis**	53.90 (± 3.50)	53.73 (± 5.80)	54.55 (± 5.54)	57.02 (49.93 to 60.44))
**Age of death**	58.78 (± 6.61)	57.36 (± 5.90)**	64.04 (± 6.58)**	59.70 (51.97 to 65.83)
**Survival years**	4.79 (± 3.50)	3.62 (± 2.12)**	9.48 (± 4.02)**	4.54 (2.33 to 6.39)*

	***N* (%)**	***N* (%)**	***N* (%)**	***N* (%)**
Sex**				
Female	91 (58)	55 (47.0)	29 (96.7)	7 (70.0)
Male	66 (42)	62 (53.0)	1 (3.3)	3 (30.0)
**Severity of ID**				
Mild	10 (6.6)	6 (5.2)	2 (6.7)	2 (33.3)
Moderate	86 (56.6)	65 (56.0)	18 (60.0)	3 (50.0)
Severe	43 (28.3)	32 (27.6)	10 (33.3)	1 (16.7)
Profound	13 (8.6)	13 (11.2)	0 (0.0)	0 (0.0)
Care setting at diagnosis**				
Specialist ID and dementia home	0	0 (0.0)	0 (0.0)	0 (0.0)
Nursing home	1 (0.6)	0 (0.0)	0 (0.0)	1 (10.0)
ID residential	40 (25.6)	17 (14.7)	14 (46.7)	9 (90.0)
Community group home	34 (21.8)	18 (15.5)	16 (53.3)	0 (0.0)
At home with relatives	81 (51.9)	81 (69.8)	0 (0.0)	0 (0.0)
**Care setting at death**† **				
Specialist ID and dementia home	30 (21.1)	0 (0.0)	30 (100.0)	‐
Nursing home	16 (11.3)	16 (14.3)	0 (0.0)	‐
ID residential	34 (23.9)	34 (30.4)	0 (0.0)	‐
Community group home	9 (6.3)	9 (8.0)	0 (0.0)	‐
At home with relatives	53 (37.3)	53 (47.3)	0 (0.0)	‐
**Comorbidities**				
LOMEDS**	115 (73.2)	90 (77.6)	23 (76.7)	2 (20.0)
Hypothyroidism*	77 (49)	51 (43.6)	21 (70.0)	5 (50.0)
Sleep apnea	12 (7.6)	10 (8.5)	2 (6.7)	0 (0.0)
Psychiatric diagnosis*	19 (12.1)	10 (8.5)	5 (16.7)	4 (40.0)
Vision impairment**	60 (38.2)	42 (35.9)	18 (60.0)	0 (0.0)
Hearing impairment*	20 (12.7)	11 (9.4)	8 (26.7)	1 (10.0)
Hypertension	4 (2.5)	2 (1.7)	2 (6.7)	0 (0.0)
Stroke/cerebrovascular	4 (2.5)	3 (2.6)	0 (0.0)	1 (10.0)
Multimorbid (2+ conditions)	115 (73.2)	81 (69.2)	27 (90.0)	7 (70.0)
AD Medication	88 (56.1)	71 (61.2)	13 (43.3)	4 (44.4)

*Note*: ^†^Data unavailable for care setting at death in IDS‐TILDA.

* *p* < 0.05

** *p* < 0.001 based on ANOVA for continuous variables and chi‐squared test for categorical variables. Data are presented as mean ± standard deviation (SD) for normally distributed variables and as median (25th–75th percentile) for non‐normally distributed variables. See Supplementary Table 1 for an expanded list of comorbidities.

Abbreviations: AD, Alzheimer's disease; DABNI, Down Alzheimer Barcelona Neuroimaging Initiative; ID, intellectual disability; IDS‐TILDA, Intellectual Disability Supplement to the Irish Longitudinal Study on Ageing.

**FIGURE 2:**
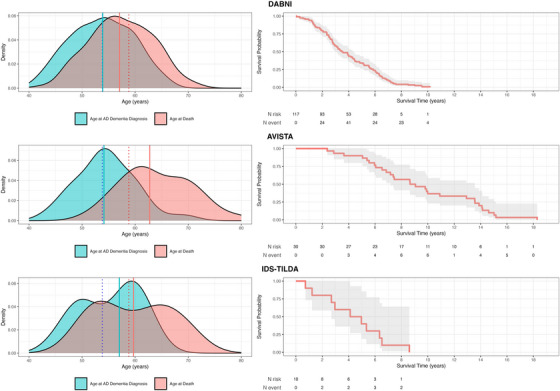
Age at diagnosis, age at death, and survival time distributions by Cohort. Left: kernel density curves for age at AD dementia diagnosis (teal) and age at death (pink); vertical solid lines mark cohort medians. The dotted line shows the average age of diagnosis and the average age at death from the meta‐analysis by Iulita et al. (2022). Right: Kaplan–Meier survival from AD dementia diagnosis to death with 95% confidence bands and numbers at risk.

When examining differences across cohorts, mean age at AD dementia diagnosis was relatively consistent, with no statistically significant difference between cohorts *F*(2, 154) = 0.50, *p* = 0.614. Mean age in DABNI was 53.7 years (SD = 5.8), in Avista 54.6 years (SD = 5.5), and in IDS‐TILDA 55.2 years (SD = 5.7). However, significant differences emerged in age at death, *F*(2, 154) = 14.40, *p* < .001, *η* ^2^ = .158, with a higher mean age at death in Avista (64.0 years, SD = 6.6) compared to DABNI (57.4 years, SD = 5.9) and IDS‐TILDA (59.6 years, SD = 7.0). Survival time also differed significantly across cohorts, *F*(2, 154) = 58.09, *p* < 0.001, *η* ^2^ = .430, with Avista showing longer mean survival (9.5 years, SD = 4.0) than DABNI (3.6 years, SD = 2.1) or IDS‐TILDA (4.5 years, SD = 2.5) (Figure 2).

Sex distributions varied significantly across cohorts, *χ^2^* (2) = 24.80, *p* < 0.001, with Avista comprising predominantly females (97%), reflecting its historical role as a women's service, compared to 47% females in DABNI and 70% in the IDS‐TILDA cohort. Care setting at AD dementia diagnosis also differed significantly, χ^2^(6) = 87.29, *p* < 0.001: While most individuals in DABNI lived at home with relatives (70%), those in Avista were primarily in ID residential (47%) or community group homes (53%), and individuals in IDS‐TILDA were predominantly in ID residential settings (90%).

Significant differences were also observed in the prevalence of comorbidities across cohorts. LOMEDS prevalence was lower in IDS‐TILDA (20%) compared to DABNI (78%) and Avista (77%), *χ* ^2^(2) = 15.92, *p* < 0.001. Hypothyroidism prevalence also differed, *χ* ^2^(2) = 6.67, *p* = 0.036, highest in Avista (70%). Rates of psychiatric diagnoses varied, *χ* ^2^ (2) = 9.29, *p* = 0.010, with IDS‐TILDA having a higher prevalence (40%) compared to DABNI (9%) or Avista (17%). Vision impairment differed significantly, *χ* ^2^(2) = 12.48, *p* = 0.002, and was highest in Avista (60%), as was hearing impairment, *χ* ^2^ (2) = 6.48, *p* = 0.039, also highest in Avista (27%). No significant differences were observed for sleep apnea, hypertension, stroke, or AD medication use across cohorts (Table 1; see Table S1 for expanded list of comorbidities).

### 3.3 Survival by demographic and clinical factors

The relationship between demographic and clinical variables and survival was examined, including age at AD dementia diagnosis, sex, level of ID, APOE status, AD medication, LOMEDS, comorbidities, and type of care setting. Unadjusted associations with survival were examined using univariate Cox proportional hazards regression; adjusted associations are from multivariable Cox models.

### 3.4 Age at AD symptom onset and age at AD dementia diagnosis

Age at AD symptom onset was available for the DABNI and Avista cohorts. Mean age at AD symptom onset was 51.5 years (SD = 5.7) in DABNI and 53.3 years (SD = 5.2) in Avista. The average time from age at AD symptom onset to AD dementia diagnosis was 2.15 years (SD = 1.80, range 0 to 8.55) in DABNI and 2.08 years (SD = 2.26, range 0 to 9.01) in Avista, with no significant difference between cohorts, *F*(1, 83) = 0.34, *p* = 0.563).

Age at AD dementia diagnosis was analyzed in the full sample (DABNI, Avista, IDS‐TILDA) across types of care settings at the time of diagnosis. Those living with relatives were diagnosed as the youngest (53.1 years; SD = 5.9) compared to 54.4 years (SD = 5.9) for those in community group homes and 55.6 years (SD = 4.8) for those in ID residential services (Table 2). A one‐way ANOVA indicated no statistically significant difference in age at AD dementia diagnosis by care setting, *F*(2, 152) = 2.86, *p* = 0.060, *η* ^2^ = 0.036, but with a trend toward earlier diagnosis for those living at home. The level of neither ID, *F*(3, 152) = 1.20, *p* = .313, nor APOE ε4 status, *F*(1, 85) = 1.07, *p* = 0.305, was significantly associated with age at AD dementia diagnosis.

**TABLE 2:** Multivariable Cox regression of survival after AD dementia diagnosis: results from a Cox proportional hazards model examining associations between listed predictors and time from AD dementia diagnosis to death. For each predictor the table reports the hazard ratio, 95% confidence interval, and *p* value. The Avista cohort was excluded.

Predictor	HR (Exp[B])	95% CI for HR	*p* value
Age at AD dementia diagnosis	1.047	[1.007, 1.089]	0.020
Sex	1.29	[0.776, 2.141]	0.327
Level of ID	1.192	[0.857, 1.658]	0.221
Care setting at death	1.030	[0.848, 1.251]	0.764
Multimorbidity	0.986	[0.603, 1.612]	0.956
AD symptom onset‐to‐LOMEDS interval	0.834	[0.749, 0.929]	<0.001
AD medication	0.845	[0.39, 1.18]	0.496

*Note*: *Avista cohort not included in model due to its unique specialist care profile and all‐female sample, which differed substantially from other cohorts. Overall model χ^2^(7) = 18.37, *p* < 0.001.

Abbreviations: AD, Alzheimer's disease; CI, confidence interval; HR, hazard ratio; ID, Intellectual Disability; LOMEDS, late‐onset myoclonic epilepsy in Down syndrome.

In the univariate Cox regression, age at AD dementia diagnosis was not significantly associated with survival time (HR per year = 1.007, 95% CI: 0.981 to 1.034, *p* = 0.60). However, in the adjusted multivariate Cox regression, higher age at AD dementia diagnosis was significantly associated with shorter survival (HR = 1.047, 95% CI: 1.01, 1.09, *p* = 0.020) (Table 2).

### 3.5 Sex

When examining the impact of sex, Avista was excluded as all except one participant were female. In the combined remaining two cohorts (*n* = 127), mean survival was 3.86 years (SD = 2.26) for females and 3.50 years (SD = 2.04) for males. In univariate Cox regression, sex was not significantly associated with survival (female vs male: HR = 1.206, 95% CI: 0.848 to 1.716, *p* = 0.30). Analyses within individual cohorts showed similarly non‐significant results for sex (Table S2). This remained non‐significant in the multivariate analysis (HR = 1.29, 95% CI: 0.78 to 2.14, *p* = 0.33).

### 3.6 Level of ID

When examining the impact of level of ID across the full sample, mean survival decreased with greater severity (mild: M = 6.07 years, SD = 4.00; moderate: M = 5.03, SD = 3.50; severe: M = 4.61, SD = 3.62; profound: M = 3.29, SD = 2.20), but the difference across levels was not statistically significant: Wald *χ* ^2^(3) = 4.99, *p* = 0.17. No significant difference was found between level of ID and survival in any of the cohorts (Table S2) and remained non‐significant in the multivariate analysis (HR = 1.19, 95% CI: 0.86, 1.66, *p* = 0.221).

### 3.7 APOE status

APOE status was available only for the DABNI cohort (*n* = 87). Within this group, the presence of the APOE ε4 allele was not significantly associated with survival time. Mean survival was 4.12 years (SD = 1.99) in ε4 non‐carriers versus 3.16 years (SD = 2.19) in ε4 carriers. In univariate Cox regression, ε4 carrier status was not significantly associated with survival (HR = 1.39, 95% CI: 0.86 to 2.27, *p* = 0.18).

### 3.8 Late‐Onset Myoclonic Epilepsy in Down syndrome

Across the combined cohorts, 73.2% (*n* = 115) were diagnosed with LOMEDS: 77.6% in DABNI, 76.7% in Avista, and 20% in IDS‐TILDA. This represented a significant difference by cohort, *χ* ^2^(2) = 15.92, *p* < 0.001. Of note, in the DABNI and Avista cohorts, the diagnoses were made by clinicians, whereas in IDS‐TILDA, data were reported from medical records. The average age of LOMEDS onset was 54.9 years (SD = 6.5; range: 40.7 to 70.8). This differed by cohort: Avista had a higher mean age at onset than DABNI (59.15, SD = 6.17 vs 53.79 SD = 6.15 years; *t*(110) = 3.66, *p* < 0.001). The average time from LOMEDS diagnosis to death was 3.9 years (SD = 2.70) across DABNI and Avista, with a significant difference in survival from LOMEDS to death between Avista (*M* = 5.65, SD = 4.14) and DABNI (*M* = 3.10, SD = 1.93); *F*(1,110) = 18.18, *p* < 0.001. Survival from LOMEDS diagnosis to death was unavailable for IDS‐TILDA.

**FIGURE 3:**
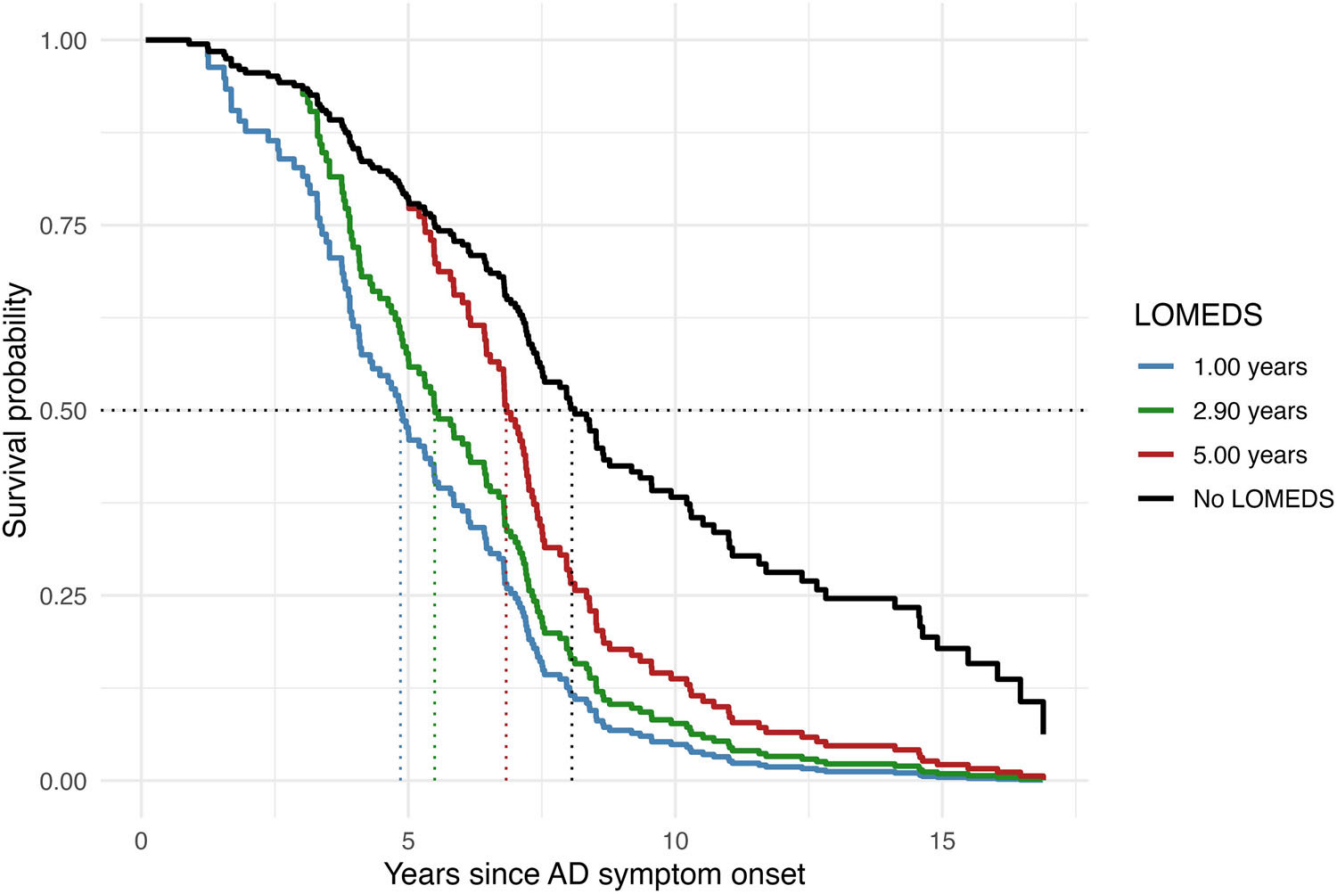
Model‐based survival under hypothetical LOMEDS onset times. Predicted survival from age at AD symptom onset for hypothetical LOMEDS onset at 1.0, 2.9 (cohort median), and 5.0 years and for never developing LOMEDS, derived from the time‐dependent Cox model (hazard ratio [HR] LOMEDS present 3.37; HR per additional latency year 0.94). Vertical dotted lines mark model‐based median survival (4.9, 5.5, 6.8, and 8.1 years, respectively). The latency effect shows a non‐significant trend (*p* = 0.073). No proportional hazards violation (global Schoenfeld test *p* = 0.52). Colors: 1 year (blue), 2.9 years (green), 5 years (red), No LOMEDS (black). AD, Alzheimer's disease; LOMEDS, late‐onset myoclonic epilepsy in Down syndrome.

A time‐dependent Cox regression model found that once LOMEDS was diagnosed, the risk of dying at any given time point was 3.37 times higher compared to when participants remained LOMEDS‐free (HR = 3.37, 95% CI: 2.15 to 5.29, *p* < 0.001). Model‐based survival curves (Figure 3) yielded a predicted median survival of 8.1 years with no LOMEDS, 6.8 years for hypothetical onset at 5 years, 5.5 years for onset at the cohort median latency (2.9 years), and 4.9 years for onset at 1 year.

Each additional year that LOMEDS onset was delayed was associated with an approximate 6% reduction in the hazard of death (HR per year = 0.94, 95% CI: 0.87 to 1.01). This represents a trend toward lower risk that did not reach conventional statistical significance (*p* = 0.073). Clinically, model predictions indicate that LOMEDS onset 5 years after the first AD symptom is predicted to confer approximately 30% longer survival compared to LOMEDS occurring at the same time as age at AD symptom onset. Model concordance was 0.65, and Schoenfeld residuals showed no violation of proportional‐hazards assumptions (global test *p* = 0.52).

### 3.9 Comorbidities

We examined the association between selected comorbidities and survival time following AD dementia diagnosis. Emotional or psychiatric conditions (HR = 0.98, 95% CI: 0.60 to 1.59, *p* = 0.93), sleep apnea (HR = 1.01, 95% CI: 0.56 to 1.83, *p* = 0.96), vision problems (HR = 0.74, 95% CI: 0.53 to 1.03, *p* = 0.08), hypertension (HR = 0.54, 95% CI: 0.20 to 1.47, *p* = 0.23), and hypothyroidism (HR = 0.76, 95% CI: 0.55 to 1.04, *p* = 0.09) were not associated with survival. These associations were also non‐significant within each individual cohort (Table S2). Hearing impairment was associated with significantly longer survival overall (HR = 0.60, 95% CI 0.37 to 0.96, *p* = 0.03), although this was not significant within individual cohorts. The combined analysis likely reflects the high prevalence of hearing impairment in Avista, influencing the results.

There was no significant difference in survival time between individuals with multimorbidity (M = 4.96 years, SD = 3.52) and those without (M = 4.31 years, SD = 3.43) in the overall sample (HR = 0.83, 95% CI: 0.58 to 1.19, *p* = 0.31) and remained non‐significant in univariate Cox analysis within cohorts (Table S2).

No significant survival difference was observed between those prescribed AD medication (M = 5.25, SD = 4.39) and those not (M = 4.55, SD = 2.75); HR = 0.81, 95% CI: 0.58 to 1.13, *p* = 0.22, and remained non‐significant when taken with other factors in the multivariate analysis. This was also non‐significant within individual cohorts (Table S2).

### 3.10 Impact of care setting on survival

When analyzed across the full sample, the type of care setting at the time of death was significantly associated with survival time from diagnosis to death: *F*(4, 137) = 22.48, *p* < 0.001 (Table 3). Mean survival was longest in those in the specialist ID and dementia home (Avista) at 9.48 years (95% CI: 7.98 to 10.99), compared to 4.13 years (95% CI: 3.33 to 4.94) in nursing home, 3.87 years (95% CI: 3.00 to 4.74) in an ID residential home, 3.79 years (95% CI: 2.57 to 5.02) in a community group home, and 3.40 years (95% CI: 2.81 to 4.00) at home with a relative.

**TABLE 3:** Mean age at AD dementia diagnosis, age at death, and survival time by care setting.

Care setting	Age at AD dementia diagnosis (mean [SD])*	Age at death (mean [SD])§	Survival time (mean [SD])§
ID and dementia specialist	n/a	64.04 [6.58]	9.48 [4.20]
Nursing home	n/a	58.37 [4.63]	4.13 [1.51]
ID residential	55.64 [4.76]	57.74 [5.99]	3.87 [2.49]
Community group home	54.35 [5.91]	58.77 [6.54]	3.79 [1.60]
At home with relative	53.07 [5.92]	56.56 [5.94]	3.40 [2.16]
**Total**	54.01 [5.71]	58.77 [6.56]	4.91 [3.59]

*Note*: *Age at AD dementia diagnosis is based on care setting at diagnosis (*n* = 154), excluding the single nursing home case. §Age at death (*n* = 142) and survival time (*n* = 141) are based on care setting at death. Shows mean values [SD] for each outcome across care settings. Age at diagnosis is grouped by care setting at diagnosis. Age at death and survival time are grouped by care setting at death. n/a indicates no cases at diagnosis in that setting.

Abbreviations: AD, Alzheimer's disease; ID, intellectual disability.

**FIGURE 4:**
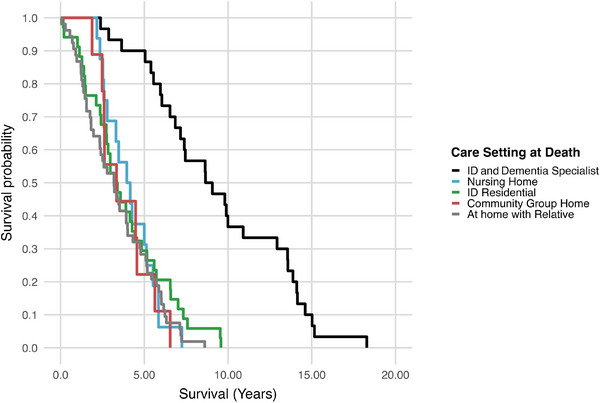
Kaplan–Meier survival curves by care setting at time of death. Time from first AD dementia diagnosis to death, stratified by care setting at death. Groups: ID and dementia specialist care (black), nursing home (blue), ID residential (green), community group home (red), at home with relative (gray). Abbreviations: AD, Alzheimer's disease; ID, intellectual disability.

To further explore these differences, Cox proportional hazards regression with care setting at death as the predictor showed that individuals in the specialist ID and dementia care setting had significantly longer survival than all other settings (*p* < 0.001), with no other pairwise differences (Table S3). Kaplan–Meier curves are presented in Figure 4 to illustrate survival patterns across settings.

## 4 DISCUSSION

This multicenter study examined factors influencing survival after AD dementia diagnosis in adults with DS across three cohorts from Spain and Ireland. Survival after dementia onset was limited and strongly influenced by both the development of LOMEDS and the post‐diagnostic care setting. Individuals who developed LOMEDS tended to have a markedly poorer prognosis, particularly when epilepsy emerged shortly after the onset of dementia. These findings provide quantitative evidence of the prognostic impact of LOMEDS in DSAD and underscore the importance of specialized dementia care environments in shaping outcomes at the end of life.

By integrating clinical and population‐based data, we also confirmed that public death certificates substantially underreport AD as the underlying cause of death in this population, which is in line with previous research.^23^, ^24^ In our cohorts, 50% of public death certificates cited congenital malformation as the underlying cause of death, whereas AD was identified as the underlying cause in 82% of cases when clinical records were used. A similar mismatch was observed when analyzing causes of death in 77,347 individuals with DS from the U.S. Centers for Disease Control and Prevention (CDC) between 1968 and 2019,^8^ where AD was attributed in slightly over 30% of certificates but was imputed in 78.9%, aligning with our data and prior work in the LonDowns cohort.^25^ This issue was compounded by 2014 CDC guidance encouraging listing DS as the underlying cause when co‐occurring with AD, contributing to persistent underreporting of AD‐related mortality.^26^, ^27^ Comparison of direct observation, clinical records, and public certificates in our study underscores that mortality patterns must be interpreted in light of ascertainment method and that clinically adjudicated data add essential clarity. Our findings further support AD as the primary driver of mortality in this population, as multimorbidity was not associated with survival when considered alongside other predictors.

Mean age at AD dementia diagnosis was consistent across cohorts and in complete agreement with a recent meta‐analytic estimate (53.9 years overall in our study vs 53.8 years in the meta‐analysis).^8^ Overall mean survival was 4.8 years, also aligning with the 4.6‐year meta‐analytic average,^8^ with a range of 3.4 to 9.5 years.

Care environment appeared to shape both detection and outcomes. Individuals living at home with relatives tended to receive an earlier diagnosis than those in formal residential settings (trend *p* = 0.06), suggesting that family caregivers may recognize subtle cognitive or behavioral changes sooner.^6^, ^25^ Although this difference did not reach conventional statistical significance, earlier recognition may still be clinically meaningful, facilitating timely access to supports and person‐centered care pathways, and these findings highlight the importance of supporting families while also strengthening early detection protocols in residential care.

The influence of the care environment on survival was even more pronounced. When analyzed across the full sample, care setting at the time of death strongly predicted the interval from diagnosis to death. Mean survival was longest for those residing in a specialist ID and dementia care setting (9.48 years) compared with 4.13 years in nursing homes, 3.87 years in ID residential settings, 3.79 years in community group homes, and 3.40 years at home with relatives. Other studies investigating survival following an AD diagnosis in individuals with DS report generally consistent median and mean survival times, typically ranging from approximately 3.8 to 4.8 years.^7^, ^8^, ^28^ Unlike previous reports that linked living at home with family to longer post‐diagnosis survival, an effect thought to be associated with an earlier age at diagnosis,^6^, ^7^ our study found that individuals cared for in a specialist ID and dementia care setting lived almost twice as long after diagnosis as those in any other care setting, including with family. Importantly, when the specialist service was removed from analyses, care setting category was no longer a significant predictor of survival, suggesting that the observed advantage reflected the specific mode of care rather than the residence type per se. Salient features of that model include trained staff with ID/dementia expertise, proactive health monitoring, regular multidisciplinary input, individualized dementia care planning, and early management of risks such as dysphagia, aspiration, and respiratory infections, well‐documented contributors to morbidity and mortality in DS.^29^, ^30^, ^31^ These data support the potential benefits of specialized services and targeted training in ID and dementia care.

Survival should also be interpreted alongside quality of life. Longer survival does not automatically imply better well‐being, particularly in advanced dementia. Best practice emphasizes comfort, proportionality, and respect for preferences, values, and dignity.^32^, ^33^ Interventions that may prolong life but add burden, such as percutaneous endoscopic gastrostomy in advanced neurodegeneration, carry complications and have not reliably improved quality of life in dementia.^33^, ^34^ By contrast, approaches that prioritize hand feeding, careful risk management for aspiration, and symptom control can better align with person‐centered goals in late‐stage disease.^33^, ^34^, ^35^, ^36^ Our finding of extended survival in specialist settings should therefore be interpreted within a broader palliative ethos, ensuring that longevity gains do not come at the expense of comfort and autonomy.

LOMEDS was highly prevalent in our decedent sample, as expected when individuals are followed until death,^12^ and is linked to worse cognitive and functional outcomes, as well as increased mortality risk after dementia onset.^16^, ^17^ Beyond prevalence, its timing carried prognostic information. Using a time‐dependent Cox model designed to avoid immortal bias, the presence of LOMEDS was associated with a 3.37‐fold higher hazard of death. Model‐based survival projections showed a clear temporal gradient: predicted median survival was 8.1 years when LOMEDS never developed, 6.8 years if onset occurred 5 years after first AD symptom, 5.5 years at the cohort median latency of 2.9 years, and 4.9 years when onset occurred 1 year after first AD symptoms. Taken together, these results indicate that earlier LOMEDS onset may mark an accelerated trajectory in DSAD and support close clinical monitoring and timely seizure management after dementia onset.^25^ While epilepsy after dementia onset has generally been considered a marker of progression in DS, prior publications did not demonstrate that the timing of LOMEDS after first AD symptom modified overall survival. Notably, a forthcoming clinical trial will explore the potential of levetiracetam to prevent seizures in this population, which may inform future approaches to managing LOMEDS in DSAD.^37^, ^38^

Previous studies identified factors such as age at AD diagnosis, severity of ID, multimorbidity, and APOE ε4 status as predictors of survival in adults with DS. Our findings partially support these associations. While the age of AD dementia diagnosis did not independently predict survival in unadjusted analyses, it emerged as a significant predictor in the multivariate Cox regression model. This suggests that the associations between older age at diagnosis and shorter survival may become more apparent when accounting for other key factors such as care setting and timing of LOMEDS onset. These findings align with those from a large UK clinical sample,^7^ which reported shorter survival among those individuals diagnosed at a later age.

We found a trend toward reduced survival among those individuals with profound versus mild ID (mean = 3.56 years, SD = 2.27 vs 6.35 years, SD = 4.00) in our cohort, in line with previous research.^7^ Individuals with DS who have more severe ID tend to live shorter lives, a pattern attributed to higher rates of epilepsy, sensory and mobility impairments, susceptibility to respiratory infections, and overall medical complexity.^1^, ^8^, ^25^, ^39^, ^40^, ^41^ The literature consistently demonstrates that individuals with DS as a group are at increased risk for respiratory infections and aspiration‐related complications due to a combination of immune dysregulation, anatomical airway differences, hypotonia, and high rates of oropharyngeal dysphagia.^30^, ^42^ However, studies specifically stratifying risk by severity of ID within the DS population are lacking.

Multiple cohort studies demonstrate that the APOE ε4 allele is associated with an earlier onset of symptomatic AD, a more rapid progression to death after dementia diagnosis, and increased overall mortality risk in this population.^15^, ^25^ We found no association between APOE status and age of AD dementia diagnosis or survival time, although this could be related to a reduced sample size as genotyping was available only in a subset of one cohort. Future studies with larger, well‐characterized DS cohorts will be essential to clarify and quantify this link between APOE ε4 and survival.

Our study has some limitations. Differences in ascertainment could have contributed to the underreporting of AD as the cause of death, particularly in the observational cohort, reflecting an inherent limitation of mortality research in this population. This work included cohorts with heterogeneous assessment methods, which could have impacted the standardization of how conditions were assessed and diagnosed. Both clinical cohorts specialized in DSAD and proactively conducted health assessments, which minimized bias. However, the observational study relied on physician‐reported diagnosis, potentially underestimating some conditions. In addition, clustering within shared residences could not be modeled, so minor residual dependence between participants might have remained. Partial APOE data further constrained inference. Future prospective studies are needed to further elucidate the mechanisms through which specialized care models and LOMEDS biology influence survival in DSAD.

## 5 CONCLUSION

The impact of post‐diagnostic dementia care on quality of life has been well documented in people with ID,^43^, ^44^, ^45^ but to our knowledge, this is the first study to show that care setting may influence survival following diagnosis of DSAD. While average survival following DSAD diagnosis appears to be consistent across previous studies,^8^ with a mean survival of approximately 5 years, this study suggests that outcomes diverge sharply by care setting and seizure profile. Residing in a specialist ID and dementia care environment nearly doubles post‐diagnostic survival, whereas the early appearance of LOMEDS signals faster decline and shorter life expectancy. These findings highlight the value of specially trained care staff, timely access to dedicated services, and vigilant epilepsy monitoring and highlight the need for larger prospective studies to unravel the genetic and neurophysiological pathways shaping survival in this population.

## AUTHOR CONTRIBUTIONS

BB, EMG, JF, and MMC were responsible for conceptualization. BB and EMG performed analysis and wrote the first draft. All authors contributed to the review and editing.

## CONFLICT OF INTEREST STATEMENT

JF reports receiving personal fees for service on the advisory boards, adjudication committees, or speaker honoraria from AC Immune, Adamed, Alzheon, Biogen, Eisai, Esteve, Fujirebio, Ionis, Laboratorios Carnot, Life Molecular Imaging, Lilly, Lundbeck, Perha, Roche, and Zambón, outside the submitted work. JF reports holding a patent for markers of synaptopathy in neurodegenerative disease (licensed to ADx, EPI8382175.0). MC‐I has received personal fees for service on advisory boards, speaker honoraria, or educational activities from IMSERSO, Esteve, Lilly, Neuraxpharm, Adium Pharma, and Roche. AL reported receiving personal fees for service on advisory boards of or speaker honoraria from Almirall, Beckman‐Coulter, Biogen, Eisai, Esteve, Fujirebio Europe, Grifols, KRKA, Lilly, Novartis, Novo Nordisk, Nutricia, Otsuka Pharmaceutical, Roche, and Zambón. AL is co‐author of a patent for markers of synaptopathy in neurodegenerative disease (licensed to ADx, EPI8382175.0) and on antibodies for amyloid precursor, methods, and uses thereof (European priority No. EP25382226). MA reported receiving speaker honoraria from Esteve Pharmaceuticals, Lilly, Neuraxpharm, Kern Pharma, Novo Nordisk, Zambon, and Nutricia. The other authors declared no potential conflicts of interest. Author disclosures are available in the Supporting Information.

## CONSENT STATEMENT

Ethical approval was obtained from the Faculty of Health Sciences Research Ethics Committee, Trinity College Dublin (FHS REC TCD), the Ethics Committee of Hospital de la Santa Creu i Sant Pau, Barcelona (Sant Pau), and the Avista Research Ethics Committee. The data related to deceased individuals, so General Data Protection Regulation did not apply; all procedures followed relevant institutional and national regulations for data on deceased persons.

## Supporting information



Supporting Information

Supporting Information

Supporting information

Supporting information
