# Supplementary material for: Factors impacting survival in individuals with Down syndrome‐associated Alzheimer's disease
Source: Alzheimers Dement. 2026 Feb 17;22(2):e71156. doi: 10.1002/alz.71156 (PMC12910243; doi:10.1002/alz.71156)
Supplement: Supplementary file 1 — Supporting Information [file ALZ-22-e71156-s001.docx]

| Supplementary Table 1: Comorbidities of Study Sample and by Cohort | | | | |
| --- | --- | --- | --- | --- |
| **Variable** | **Total Sample**  **(n = 157)** | **DABNI**  **(n = 117)** | **AVISTA**  **(n = 30)** | **IDS-TILDA**  **(n = 10)** |
|  | **N (%)** | **N (%)** | **N (%)** | **N (%)** |
| LOMEDS | 115 (73.2) | 90 (77.6) | 23 (76.7) | 2 (20.0) |
| Hypothyroidism | 77 (49.0) | 51 (43.6) | 21 (70.0) | 5 (50.0) |
| Vision Impairment | 60 (38.2) | 42 (35.9) | 18 (60.0) | 0 (0.0) |
| Psychiatric Diagnosis | 19 (12.1) | 10 (8.5) | 5 (16.7) | 4 (40.0) |
| Hearing Impairment | 20 (12.7) | 11 (9.4) | 8 (26.7) | 1 (10.0) |
| Diabetes | 16 (10.2) | 13 (11.1) | 2 (6.7) | 1 (10.0) |
| Degenerative joint disease (arthritis) | 14 (8.9) | 8 (6.8) | 6 (20.0) | 0 (0.0) |
| Peripheral autoimmune condition | 13 (8.3) | 7 (6.0) | 6 (20.0) | 0 (0.0) |
| Obstructive sleep apnea | 12 (7.6) | 10 (8.5) | 2 (6.7) | 0 (0.0) |
| Hypercholesterolaemia | 13 (8.3) | 6 (5.1) | 6 (20.0) | 1 (10.0) |
| Congenital heart disease | 13 (8.3) | 12 (10.3) | 0 (0.0) | 1 (10.0) |
| Osteoporosis or osteopenia | 13 (8.3) | 2 (1.7) | 9 (30.0) | 2 (20.0) |
| Constipation | 13 (8.3) | 3 (2.6) | 7 (23.3) | 3 (30.0) |
| Liver disease | 10 (6.4) | 9 (7.7) | 1 (3.3) | 0 (0.0) |
| Kidney disease | 11 (7.0) | 6 (5.1) | 4 (13.3) | 1 (10.0) |
| Chronic inflammatory skin disease (psoriasis) | 9 (5.7) | 6 (5.1) | 3 (10.0) | 0 (0.0) |
| Thrombosis | 8 (5.1) | 6 (5.1) | 2 (6.7) | 0 (0.0) |
| Dental inflammation | 9 (5.7) | 6 (5.1) | 1 (3.3) | 2 (20.0) |
| Chronic respiratory disease | 8 (5.1) | 6 (5.1) | 1 (3.3) | 1 (10.0) |
| Asthma | 5 (3.2) | 4 (3.4) | 0 (0.0) | 1 (10.0) |
| Hypertension | 4 (2.5) | 2 (1.7) | 2 (6.7) | 1 (10.0) |
| Stroke / Cerebrovascular | 4 (2.5) | 3 (2.6) | 0 (0.0) | 1 (10.0) |
| Peripheral vascular disease | 3 (1.9) | 3 (2.6) | 0 (0.0) | 0 (0.0) |
| Cancer (solid tumour) | 3 (1.9) | 3 (2.6) | 0 (0.0) | 0 (0.0) |
| Other endocrine disorders | 2 (1.3) | 2 (1.7) | 0 (0.0) | 0 (0.0) |
| Ischaemic heart disease | 2 (1.3) | 2 (1.7) | 0 (0.0) | 0 (0.0) |
| Haematological malignancy | 1 (0.6) | 0 (0.0) | 1 (3.3) | 0 (0.0) |

NOTE: Complete list of comorbidities for the total study sample and by cohort. For the pooled analyses, only the following comorbidities were included: hypothyroidism, sleep apnea, psychiatric diagnosis, vision impairment, hearing impairment, hypertension and stroke or cerebrovascular disease.
