# Supplementary material for: Factors impacting survival in individuals with Down syndrome‐associated Alzheimer's disease
Source: Alzheimers Dement. 2026 Feb 17;22(2):e71156. doi: 10.1002/alz.71156 (PMC12910243; doi:10.1002/alz.71156)
Supplement: Supplementary file 2 — Supporting Information [file ALZ-22-e71156-s003.docx]

| Supplementary Table 2: Factors associated with survival time by Cohort | | | | | | | | | |
| --- | --- | --- | --- | --- | --- | --- | --- | --- | --- |
|  | **DABNI** | | | **AVISTA** | | | **IDS-TILDA** | | |
| **Variable** | **n (%)** | **Mean survival (SD)** | **p-value** | **n (%)** | **Mean survival (SD)** | **p-value** | **n (%)** | **Mean survival (SD)** | **p-value** |
| **Sex** |  |  |  |  |  |  |  |  |  |
| Female | 55 (47.6) | 3.91 (2.27) | 0.170 | 29 (96.7) | 9.73 (4.05) | 0.086 | 7 (70.0) | 3.51 (2.29) | 0.106 |
| Male | 62 (52.4) | 3.37 (1.96) |  | 1 (3.3) | 2.38 (NA) |  | 3 (30.0) | 6.30 (2.00) |  |
| **Level of ID** |  |  |  |  |  |  |  |  |  |
| Mild | 6 (5.2) | 4.70 (2.72) | 0.234 | 2 (6.7) | 12.51 (3.56) | 0.684 | 2 (20.0) | 5.16 (0.27) | 0.106 |
| Moderate | 65 (56.0) | 4.41 (2.20) |  | 18 (60.0) | 9.44 (4.15) |  | 3 (30.0) | 1.55 (1.02) |  |
| Severe | 32 (27.6) | 3.36 (2.11) |  | 10 (33.3) | 8.95 (4.54) |  | 4 (40.0) | 5.50 (2.50) |  |
| Profound | 13 (11.2) | 3.57 (2.27) |  | - | - |  | 1 (10.0) | 6.52 (NA) |  |
| **Hypothyroidism** |  |  |  |  |  |  |  |  |  |
| Yes | 52 (44.4) | 4.05 (2.16) | 0.118 | 21 (70.0) | 8.49 (3.68) | 0.123 | 5 (50.0) | 4.23 (2.26) | 0.837 |
| No | 65 (55.6) | 3.28 (2.04) |  | 9 (30.0) | 11.80 (4.63) |  | 5 (50.0) | 4.47 (2.97) |  |
| **Emotional or Psychiatric Condition** |  |  |  |  |  |  |  |  |  |
| Yes | 10 (8.6) | 3.03 (2.16) | 0.501 | 5 (16.7) | 9.13 (5.52) | 0.874 | 4 (40.0) | 3.91 (1.86) | 0.348 |
| No | 107 (91.4) | 3.68 (2.12) |  | 25 (83.3) | 9.55 (4.03) |  | 6 (60.0) | 4.64 (2.97) |  |
| **Vision impairment** |  |  |  |  |  |  |  |  |  |
| Yes | 42 (36.2) | 3.50 (1.76) | 0.267 | 18 (60.0) | 10.18 (4.30) | 0.367 | 0 (0.0) | NA | - |
| No | 75 (63.8) | 3.69 (2.31) |  | 12 (40.0) | 8.43 (4.00) |  | 10 (100.0) | 4.35 (2.49) |  |
| **Hearing impairment** |  |  |  |  |  |  |  |  |  |
| Yes | 11 (9.5) | 4.73 (2.71) | 0.061 | 8 (26.7) | 9.95 (2.65) | 0.845 | 1 (10.0) | 0.73 (NA) | 0.132 |
| No | 106 (90.5) | 3.51 (2.03) |  | 22 (73.3) | 9.31 (4.68) |  | 9 (90.0) | 4.75 (2.27) |  |
| **Multimorbidity** |  |  |  |  |  |  |  |  |  |
| Yes | 81 (69.8) | 3.73 (2.21) | 0.340 | 27 (90.0) | 9.07 (3.94) | 0.115 | 7 (70.0) | 3.37 (2.11) | 0.131 |
| No | 36 (31.0) | 3.37 (1.92) |  | 3 (10.0) | 13.18 (5.62) |  | 3 (30.0) | 6.64 (1.83) |  |
| **APOE ε4 status** |  |  |  |  |  |  |  |  |  |
| Carrier | 23 (26.4) | 3.16 (2.19) | 0.182 | - | - | - | - | - | - |
| Non-carrier | 64 (73.6) | 4.12 (1.99) |  | - | - |  | - | - |  |

**Legend:** This table presents the number (%) of participants across three cohorts: DABNI (Hospital de la Santa Creu i Sant Pau, Barcelona, Spain), AVISTA CLG, Dublin, Ireland, and IDS-TILDA (Intellectual Disability Supplement to The Irish Longitudinal Study on Ageing, Ireland). Mean (SD) refers to mean survival years (time from AD Dementia diagnosis to death) with standard deviation. p-values represent overall scores test in univariate Cox regression. Estimates for IDS_TILDA should be interpreted with caution due to small n. APOE ε4 data were available only for the DABNI cohort.
