# Supplementary material for: Factors impacting survival in individuals with Down syndrome‐associated Alzheimer's disease
Source: Alzheimers Dement. 2026 Feb 17;22(2):e71156. doi: 10.1002/alz.71156 (PMC12910243; doi:10.1002/alz.71156)
Supplement: Supplementary file 3 — Supporting information [file ALZ-22-e71156-s004.docx]

| Supplementary Table 3: Survival comparisons between care settings at death in individuals with Down syndrome and Alzheimer’s disease | | | | | |
| --- | --- | --- | --- | --- | --- |
| **Reference ↓ vs Comparison →** | **ID & Dementia Specialist** | **Nursing Home** | **ID Residential** | **Community Group Home** | **At home with Relative** |
| **ID & Dementia Specialist** | ref | **HR=6.65, p<.001** | **HR=5.81, p<.001** | **HR=7.55, p<.001** | **HR=7.80, p<.001** |
| **Nursing Home** | **HR=0.15, p<.001** | ref | HR=0.87, p=.661 | HR=1.14, p=.760 | HR=1.17, p=.578 |
| **ID Residential** | **HR=0.17, p<.001** | HR=1.15, p=.661 | ref | HR=1.30, p=.489 | HR=1.34, p=.189 |
| **Community Group Home** | **HR=0.13, p<.001** | HR=0.88, p=.760 | HR=0.77, p=.489 | ref | HR=1.03, p=.930 |
| **At home with Relative** | **HR=0.13, p<.001** | HR=0.85, p=.578 | HR=0.75, p=.189 | HR=0.97, p=.930 | ref |

**Legend.** Values represent hazard ratios (HR) from Cox proportional hazards regression with each care setting as the reference category in separate models. HR > 1 indicates shorter survival relative to the reference group; HR < 1 indicates longer survival. Bolded p-values indicate statistical significance at p < .05. All models used survival time from Alzheimer’s disease diagnosis to death as the dependent variable and care setting at death as the predictor.
